# Unveiling Loneliness in Multiple Sclerosis: Insights from a Nationwide Cross-Sectional Study in Greece

**DOI:** 10.1192/j.eurpsy.2025.871

**Published:** 2025-08-26

**Authors:** M. Bakola, A. K. Sakaretsanou, K. S. Kitsou, N. Vaitsis, S. Aggelakou-Vaitsi, M. E. G. Elsayed, K. Argyropoulos, P. Gourzis, E. Jelastopulu

**Affiliations:** 1 Department of Public Health, University of Patras, Medical School, Patras, Greece; 2 Department of Psychiatry, Carl von Ossietzky University Oldenburg, School of Medicine and Health Sciences, Oldenburg, Germany; 3 Department of Psychiatry, University of Patras, Medical School, Patras, Greece

## Abstract

**Introduction:**

Multiple sclerosis (MS) is a chronic and progressive inflammatory autoimmune disease of the central nervous system. Beyond physical symptoms, it can cause various socio‐affective symptoms such as depression, anxiety, sleep disorders and loneliness, leading to a significant psychosocial burden.

**Objectives:**

This study aimed to identify factors contributing to loneliness in MS patients and to examine its associations with psychological distress, stigma, and resilience.

**Methods:**

We conducted a nationwide cross-sectional study of patients with MS from October 2022 to January 2023. Data were collected using an online questionnaire, which included socio-demographic information, disease characteristics, experiences of social stigma, psychological distress, coping strategies, and perceived social support. Validated tools used were the Stigma Scale of Chronic Illness (SSCI-8), Kessler Psychological Distress Scale (K10), Brief Resilient Coping Scale (BRCS), and UCLA Loneliness Scale.

**Results:**

A total of 108 patients, 69.4% women, mean age 44.8 years, participated in the study. Higher loneliness scores were associated with greater psychological distress (p<0.001) and higher perceived stigma (p<0.001). Inversely, higher loneliness levels correlated with lower resilience (p<0.001). Patients living in small urban or rural areas reported higher levels of loneliness compared to those in large urban centers (p=0.002). Additionally, full-time employment (p=0.032) and better financial status (p = 0.025) were associated with reduced loneliness, while a family history of psychiatric illness was linked to higher loneliness (p=0.043).

**Conclusions:**

This study reveals that loneliness is an important issue in MS patients and is associated with mental health problems, stigma and reduced coping resilience. Patients living in smaller urban areas, with poorer financial status, or a family history of psychiatric illness are particularly vulnerable. Addressing loneliness should be a priority in psychosocial interventions to improve quality of life. Future research with larger samples is recommended to confirm and extend these findings.

**Disclosure of Interest:**

None Declared

